# Antibiotic-associated dysbiosis affects the ability of the gut microbiota to control intestinal inflammation upon fecal microbiota transplantation in experimental colitis models

**DOI:** 10.1186/s40168-020-00991-x

**Published:** 2021-02-06

**Authors:** Francesco Strati, Meritxell Pujolassos, Claudia Burrello, Maria Rita Giuffrè, Georgia Lattanzi, Flavio Caprioli, Jacopo Troisi, Federica Facciotti

**Affiliations:** 1 Department of Experimental Oncology, IEO European Institute of Oncology IRCCS, Milan, Italy; 2 Theoreo srl, Spin-off Company of the University of Salerno, Montecorvino Pugliano, Italy; 3 Department of Pathophysiology and Transplantation, Università degli Studi di Milano, Milan, Italy; 4 Gastroenterology and Endoscopy Unit, Fondazione IRCCS Cà Granda, Ospedale Maggiore Policlinico, Milan, Italy; 5 European Biomedical Research Institute of Salerno (EBRIS), Salerno, Italy

**Keywords:** FMT, IBD, Antibiotics, Gut microbiota, iNKT

## Abstract

**Background:**

The gut microbiota plays a central role in host physiology and in several pathological mechanisms in humans. Antibiotics compromise the composition and functions of the gut microbiota inducing long-lasting detrimental effects on the host. Recent studies suggest that the efficacy of different clinical therapies depends on the action of the gut microbiota. Here, we investigated how different antibiotic treatments affect the ability of the gut microbiota to control intestinal inflammation upon fecal microbiota transplantation in an experimental colitis model and in ex vivo experiments with human intestinal biopsies.

**Results:**

Murine fecal donors were pre-treated with different antibiotics, i.e., vancomycin, streptomycin, and metronidazole before FMT administration to colitic animals. The analysis of the gut microbiome, fecal metabolome, and the immunophenotyping of colonic lamina propria immune cells revealed that antibiotic pre-treatment significantly influences the capability of the microbiota to control intestinal inflammation. Streptomycin and vancomycin-treated microbiota failed to control intestinal inflammation and were characterized by the blooming of pathobionts previously associated with IBD as well as with metabolites related to the presence of oxidative stress and metabolism of simple sugars. On the contrary, the metronidazole-treated microbiota retained its ability to control inflammation co-occurring with the enrichment of *Lactobacillus* and of innate immune responses involving iNKT cells. Furthermore, ex vivo cultures of human intestinal lamina propria mononuclear cells and iNKT cell clones from IBD patients with vancomycin pre-treated sterile fecal water showed a Th1/Th17 skewing in CD4^+^ T-cell populations; metronidazole, on the other hand, induced the polarization of iNKT cells toward the production of IL10.

**Conclusions:**

Diverse antibiotic regimens affect the ability of the gut microbiota to control intestinal inflammation in experimental colitis by altering the microbial community structure and microbiota-derived metabolites.

Video Abstract

**Supplementary Information:**

The online version contains supplementary material available at 10.1186/s40168-020-00991-x.

## Background

The human gut microbiota plays a crucial role in maintaining the integrity of the GI tract, the immune system homeostasis, and the host energy metabolism. Alterations of this network can lead to pathological abnormalities and inflammation [1]. Antibiotics revolutionized medicine regarding the treatment of infectious diseases worldwide; however, their effects are not limited to pathogens for which they are prescribed and indiscriminately affect also the growth of beneficial microbes, including those residing in the gut [2]. By decreasing the diversity of the microbiota, antibiotics compromise host-microbes interactions, immune system homeostasis [2], and impair colonization resistance toward incoming pathogenic bacteria [3]. Indeed, antibiotic treatments worsen the severity of dextran sodium sulfate (DSS)-induced colitis, a mouse model of inflammatory bowel diseases (IBD), through the disturbance of toll-like receptors (TLRs) signaling [4]. Antibiotic-induced dysbiosis promote also sustained T cell-mediated dysfunctions and increased susceptibility to inflammation and infections by interfering with microbiota-dependent regulation of intestinal innate immunity [5]. Accordingly, we showed that short-term broad-spectrum oral antibiotic treatment profoundly affects the phenotype and function of mucosal immune cell populations by inducing inflammatory activation of colonic iNKT and T helper cells [6]. These studies highlight the important implications of repeated broad-spectrum antibiotic use and the breakdown of tolerance mechanisms between the microbiota and the immune system. Strong epidemiological evidences link antibiotic usage early in life with an increased risk of inflammatory conditions such as IBD, indicating an important role for the gut microbiota in modulating intestinal immunity [7, 8]. It has been proposed that the effects of antibiotics on gut microbiota composition and host physiology may relate to their mechanism of action. For instance, vancomycin, which is a Gram^+^ targeting drug [9] affects also Gram^−^ commensals causing long-lasting susceptibility to a variety of secondary infections [10]. On the contrary, low-dose metronidazole is associated with a decreased rate of endoscopic recurrence of Crohn’s disease after ileal resection [11].

Presently, little is known about the interaction between the gut microbiota, their metabolites, and relevant inflammatory responses in the gut and the impact on host immunity. We recently showed that control of experimental intestinal inflammation by fecal microbiota transplantation (FMT) is dependent on the transfer of a core microbial ecology composed by different bacterial taxa (*Bacteroidales S24-7*, *Lachnospiraceae*, *Lactobacillaceae*, *Ruminococcaceae*, *Rikenellaceae*, *Bifidobacteriaceae*, and *Erysipelotrichaceae*) sharing similar metabolic functions (i.e., SCFA production, Ph control, free radicals scavenging) [12, 13]. We showed that when this core microbiota is maintained at a certain population level, it creates environmental conditions sufficient to inhibit the growth of pathogenic species/pathobionts, shapes host mucosal immune cell functions, and supports optimal host health [13]. Indeed, transfer of a gut microbiota enriched in these bacterial taxa during acute and chronic experimental colitis is capable to modulate directly both innate and adaptive mucosal immune responses by inducing IL10-dependent anti-inflammatory responses [12, 13].

Here, we investigated how antibiotics-conditioned gut microbiota modulates the disease outcome in a model of experimental colitis upon FMT. Amplicon-based 16S rRNA gene analysis of the gut microbiota, fecal metabolomics, and immunophenotyping of colonic lamina propria (LP) immune cells in recipient colitic animals were analyzed. Reciprocal functional interactions between the microbiota, the metabolome, and the mucosal immune system were analyzed to identify key elements fundamental for the restoration of a healthy gut microenvironment and the re-instruction of the immune system toward homeostasis. In vitro/ex vivo experiments in human settings further confirmed our findings.

Taken together, our results shed lights on the effects that alteration of the gut microbiota composition, e.g., by antibiotic treatment, may have on the gut microbiota and its capacity to induce or control intestinal inflammation. These findings may be of pivotal importance in those therapeutic settings in which restoration of homeostatic host-microbes interactions and immune functions are required for the stable management of inflammatory disease.

## Methods

### Mice

C57BL/6 mice (Charles River, IT) of 8-10 weeks of age were housed at the IEO animal facility in SPF conditions. Experimental groups of mice receiving FMT treatments were kept in separate cages. Littermates of the same sex and age were randomly assigned to the different experimental groups. Animal experimentations were approved by the Italian Ministry of Health (Auth. 415/2017) and by the animal welfare committee (OPBA) of the European Institute of Oncology (IEO), Italy.

### Experimental colitis model and FMT treatments

For the induction of DSS-mediated acute colitis, mice were given 2% (w/v) dextran sodium sulfate (DSS, MW 40 kD; TdB Consultancy) in their drinking water for 7 days followed by 2 days of recovery. At sacrifice, colons were collected, their length was measured and divided in portions to isolate lamina propria mononuclear cells (LPMC). FMT was performed through oral gavage of mucus (first day) and feces (second and third days) preparations from donor mice as previously described [13]. Microbiota for FMT was obtained from untreated (normobiotic) mice or mice treated with metronidazole (1 g/L), vancomycin (1 g/L), or streptomycin (2 g/L). Mucus was scraped from colons, diluted in PBS, and administered to recipients at 1:1 ratio. Feces were collected, diluted in PBS (50 mg/ml), and administered to recipients by oral gavage (10 mg/mouse) 1 day after the end of acute DSS administration to recipient animals.

### Inflammation immunoscore

Clinical (colon length) and immunological (proinflammatory genes expression, CD4^+^T colonic infiltrate and IL10 protein level) parameters were evaluated in colitic mice undergoing or not FMT treatment. A scoring system has been generated and used to evaluate the inflammation status of mice in 5 independent experiments. Points were recorded according to the following scale: Colon length, > 7 cm (0), 6-6.9 cm (1), < 5.9 cm (2); proinflammatory gene expression (*Il1b* and *Tnf*), < 0.0099 (0), 0.01-0.099 (1), > 0.099 (2); CD4^+^T cell infiltrate (abs numbers and % ki67^+^), < 5000 and < 10% (0), 5000-15000 and 10-20% (1), > 15000 and > 20% (2); IL10 protein expression (pg), > 20000 (0), 10000-20000 (1), < 10000 (2).

### Murine cell isolation

Single-cell suspensions were prepared from the colon of C57BL/6 as previously described [13]. LPMCs were isolated from colons upon incubation with 5 mM EDTA at 37 °C for 30 min, followed by further digestion with collagenase IV and DNase at 37 °C for 1 h. Cells were then separated with a Percoll gradient. After isolation, some cells were re-stimulated in vitro for 3 h with PMA/ionomycin in the presence of Brefeldin A for cytokine secretion.

### RT-qPCR of tissue mRNA

Total RNA from colonic tissues was isolated as previously described [13]. cDNAs were generated from 1 μg of total RNA with reverse transcription kit (Promega). Gene expression levels were evaluated by qPCR and normalized to *Rpl32* gene expression.

### 16S rRNA gene sequencing and data analysis

DNA extraction, 16S rRNA gene amplification, purification, library preparation, and pair-end sequencing on the Illumina MiSeq platform were performed as described in [13]. Reads were pre-processed using the MICCA pipeline (v.1.5) (http://www.micca.org) [14]. Forward and reverse primers trimming and quality filtering were performed using *micca trim* and *micca filter*, respectively. De novo greedy clustering and chimera filtering were performed by using *micca otu*: operational taxonomic units (OTUs) were assigned by clustering the sequences with a threshold of 97% pairwise identity, and their representative sequences were taxonomically classified using *micca classify* with the RDP classifier [15]. Multiple sequence alignment (MSA) of 16S rRNA gene sequences was performed using the Nearest Alignment Space Termination (NAST) [16] algorithm implemented in *micca msa* with the template alignment clustered at 97% similarity of the Greengenes database [17] (release 13_05). Phylogenetic trees were inferred using *micca tree* [18]. Sampling heterogeneity was reduced rarefying samples at the depth of the less abundant sample using *micca tablerare*. *Alpha* (within-sample richness) and *beta*-diversity (between-sample dissimilarity) estimates were computed using the phyloseq R package [19]. Permutational multivariate analysis of variance (PERMANOVA) test was performed using the adonis() function in the R package vegan with 999 permutations. Linear discriminant effect size analysis (LEfSe) was performed to find features (microbial taxa) most likely to explain differences between classes [20]. Random Forest [21] analyses of 16S rRNA gene sequencing data were performed using the randomForest R package; permutation tests with 1000 permutations were performed to assess model significance [22].

### Metabolomic analysis

Metabolome extraction, purification, and derivatization were carried by means of the MetaboPrep kit (Theoreo srl, Montecorvino Pugliano, Salerno, Italy) according to the manufacturer’s instruction. Two-microliter samples of the derivatized solution were injected into the GC-MS system (GC-2010 Plus gas chromatograph coupled to a 2010 Plus single quadrupole mass spectrometer; Shimadzu Corp., Kyoto, Japan). Chromatographic separation was achieved as previously reported [13, 23] using a 30 m 0.25 mm CP-Sil 8 CB fused silica capillary GC column with 1.00 μm film thickness from Agilent (Agilent, J&W Scientific, Folsom, CA, USA), with helium as carrier gas. Untargeted metabolites were identified by comparing the mass spectrum of each peak with the NIST library collection (NIST, Gaithersburg, MD, USA). To identify metabolites identity, the linear index difference max tolerance was set at 10, while the minimum matching for the NIST library search was set at 85%. According to MSI level 1 standard [24], the relevant putative metabolites identity was further confirmed by means of an independent analytical standard analysis. The normalization procedures consisted of data transformation and scaling. Data transformation was made by generalized log transformation and data scaling by autoscaling (mean-centered and divided by the standard deviation of each variable). To analyze metabolomic data, partial least square discriminant analysis (PLS-DA) [25] was performed on Internal Standard peak area [26] normalized chromatogram using R. Mean centering and unit variance scaling were applied for all analyses. Classification and cross-validation were performed using the wrapper function included in the caret package. A permutation test was performed to assess the significance of class discrimination. Variable importance in projection (VIP) scores were calculated for each component. To identify the most meaningful changes in two conditions, the volcano plot was built by plotting the negative log of the *p* value on the *y* axis.

### Antibiotic treatment of stool samples and fecal water preparation

Stool samples from three adult healthy donors were resuspended 1:10 (w/v) in pre-reduced PBS + 10% FBS and filtered through a 0.75 μm filter to remove large debris. The fecal slurries were then incubated with metronidazole (333 μg/ml) streptomycin (333 μg/ml) and vancomycin (125 μg/ml), respectively, at 37 °C in anaerobiosis for 8 h. After the antibiotic treatments, samples were centrifuged at 13.000 rpm × 15′ and sterile filtered through a 0.2-μm filter to obtain the sterile fecal water samples. Before fecal water preparation, the antibiotic-treated fecal slurries were plated on GAM agar at 37 °C in anaerobiosis for 72 h for bacterial cell count.

### Ex vivo LPMC stimulation assay

Human LPMCs were isolated from specimens of IBD patients undergoing intestinal surgical resection as previously described [27]. The study protocol was approved by the ethics committee (Comitato Etico Milano Area 2) of the Fondazione IRCCS Cà Granda, Ospedale Maggiore Policlinico, Milan, Italy (ref #557_2018). Briefly, the dissected intestinal mucosa was freed of mucus and epithelial cells in sequential steps with DTT (0.1 mmol/l) and EDTA (1 mmol/l) (both from Sigma-Aldrich) and then digested with collagenase D (400 U/ml) (Worthington Biochemical Corporation) for 5 h at 37 °C. LPMCs were then separated with a Percoll gradient and cultured in complete RPMI 1640 medium containing 5% human serum (Sigma-Aldrich) and 100 U/ml IL-2 (Proleukin). LPMCs were plated at a density of 2 × 10^5 ^and treated with the fecal water samples (1:5 v/v, fecal water:cells) for 24 h. At the end of the experiment, cells were analyzed with flow cytometry and supernatants were collected for ELISA assays. Cell viability was checked with Zombie Yellow™ Fixable Viability Kit (Biolegend).

### iNKT cell clones generation and in vitro stimulation

Human iNKT cell lines were generated from sorted CD45^+^CD3^+^CD1d:PBS57Tet^+^ cells isolated from total LPMCs as previously described [28]. Sorted iNKT cells were expanded in vitro for 2 weeks in the presence of irradiated peripheral blood feeders, hIL2 (100 U/ml; Proleukin), and PHA (1 μg/ml; Sigma-Aldrich). iNKT cell clones were generated via cloning by limiting dilution according to the protocol described in [29]. For fecal water stimulation, 5 × 10^4^ THP1 cells were plated in each well in 1:1 ratio with human iNKT cells and treated with the fecal water samples (1:5 v/v, fecal water:cells) for 24 h. αGalCer was used at 100 ng/ml as control for iNKT cells stimulation.

### Flow cytometry

Human and murine LPMC were stained with labeled antibodies diluted in PBS with 1% heat-inactivated fetal bovine serum (FBS) for 20 min on ice. Intracellular staining of cytokines was performed after cell fixation and permeabilization with cytofix/cytoperm (BD) before addition of the antibodies. Samples were analyzed with a FACSCelesta flow cytometer (BD Biosciences, Franklin Lakes NJ, USA). The antibodies and dyes used in the study are reported in Table S1. Data were analyzed using the FlowJo software (TreeStar, Ashland, OR, USA) or FACS Diva software (BD Biosciences, Franklin Lakes NJ, USA).

### ELISA assay

Cytokines from LPMC supernatants were detected by coating with anti-human capturing antibody for IL17, IFNɣ, TNFα, IL6, and IL10 (Biolegend) overnight at 4 °C. Blocking of the plates was performed with PBS/BSA 1% for 1 h at room temperature, while sample incubation was performed overnight at 4 °C. Standards and samples were analyzed in duplicates. Protein detection was performed with 1 μg/ml of biotin anti-human detection antibody for IL17, IFNɣ, TNFα, IL6, or IL10 (Biolegend) for 2 h at room temperature. Streptavidin-HRP conjugate (Biolegend) and TMB chromogen solution (ThermoFisher) were used to develop the assay, according to manufacturer’s instruction. Reaction was blocked using sulfuric acid 1 N. Plates were read at a wavelength of 450 nm. Murine colonic tissues were homogenized in 300 μl RIPA buffer (Cell Signaling Technology) supplemented with phosphatase inhibitors (Sigma) and protease inhibitors (Complete Ultra tablets, Roche). The samples were then incubated at 4 °C for 30 min under slow rotation and then centrifuged at 13000 rpm for 15 min at 4 °C. The supernatant was quantified at the NanoDrop with Bradford Assay (BioRad). mIL10 was measured on 6.25 μg of lysate using an ELISA assay (Purified anti-mouse IL10 and Biotin anti-mouse IL10, Biolegend) performed following manufacturer’s instructions.

### Statistical analysis

Statistical analyses were performed using R [30] and GraphPad Prism v8.3.1 (GraphPad Software, La Jolla, CA, USA). At least two independent replicates have been performed per each experiment. Data were represented as boxplot, i.e., with the lower and upper hinges corresponding to the first and third quartiles (the 25th and 75th percentiles). The upper and lower whiskers extend no further than ± 1.5×IQR from the hinges. Outliers were plotted individually. Otherwise, data were represented as barplot with mean ± standard error of the mean (SEM). The Wilcoxon rank-sum test was used for the comparison between groups, and the resulting *p* values were corrected for multiple testing controlling the false discovery rate (FDR) [31] if not stated otherwise. Spearman’s correlation tests were computed using the *psych* R package [32] and *p* values were FDR-corrected [31] for multiple comparisons. No data were excluded from analyses.

## Results

### Antibiotics pre-conditioning affects the ability of the gut microbiota to control intestinal inflammation upon FMT

**Fig. 1:**
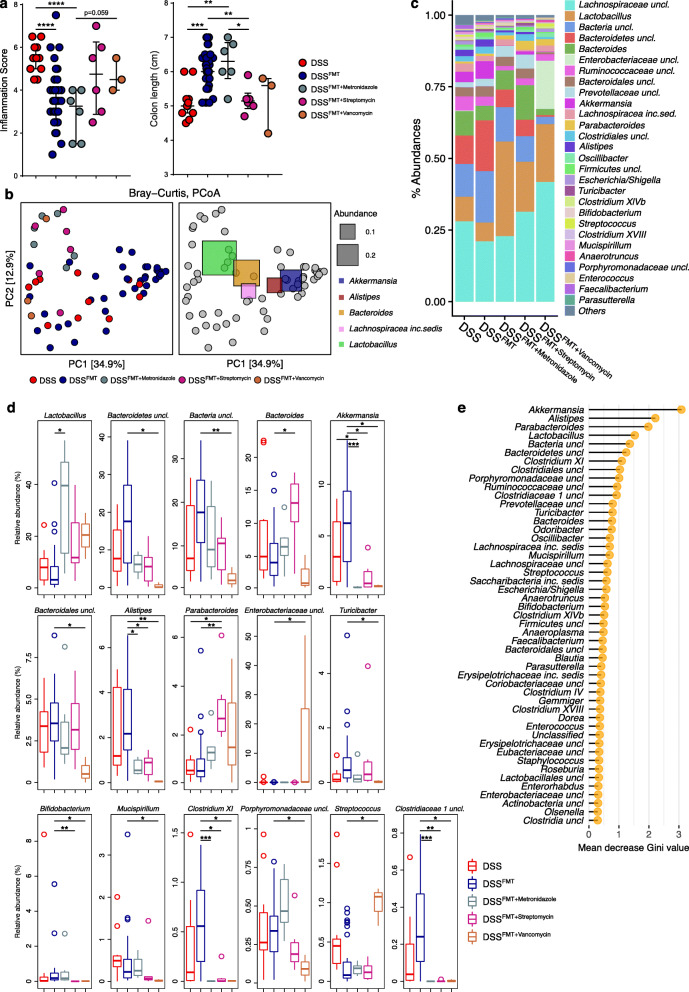
Experimental colitis outcome and gut microbiota composition upon FMT with antibiotics pre-conditioned microbiota in DSS colitic animals. **a** Experimental colitis outcome as measured by inflammation scores and colon length. **b** PCoA of beta-diversity as measured by Bray-Curtis dissimilarity. In the right panel, PCoA generated as in the left panel showing the five most abundant, fully classified, genera superimposed as colored squares, with size being proportional to the mean relative abundance of the taxon across all samples (in gray). **c** Mean relative abundance at genus level of the gut microbiota among groups. All genera with relative abundance < 0.1% are reported together and labeled as “others.” **d** Most abundant genera (with relative abundance > 0.1%) showing significant differences in their relative abundance among groups. *P* values were FDR corrected. *p < 0.05, **p < 0.01, *** < 0.005; Wilcoxon rank-sum test. **e** Random forest analysis. Bacterial taxa with the highest discriminatory power sorted by mean decrease GINI value

The composition of the gut microbiota transferred into colitic recipients influence FMT success rate [33]. To understand the impact of gut microbiota alterations on inflammatory conditions, wild-type SPF murine stool donors were pre-treated with antibiotics having different mechanisms of action, i.e., metronidazole, streptomycin, and vancomycin, before FMT administration to recipient colitic animals. The effect of the treatment on disease outcome was evaluated by using a scoring system accounting for clinical and immunological parameters (see the “Methods” section). Pre-conditioned metronidazole FMT-treated mice (DSS^FMT + Metronidazole^ mice) showed reduced signs of intestinal inflammation similar to mice treated with normobiotic fecal samples (DSS^FMT^ mice), as indicated by decreased inflammation score and increased colon length compared to untreated colitic mice (DSS mice) (Fig. 1a). On the contrary, pre-conditioned streptomycin and vancomycin FMT-treated mice (DSS^FMT + Streptomycin^ and DSS^FMT + Vancomycin^ mice, respectively) showed clear signs of intestinal inflammation, similar to those observed in DSS mice (Fig. 1a). Notably, half of DSS^FMT + Vancomycin^ animals in the study were sacrificed before the experimental endpoint because of colitis severity. To track the modifications of the gut microbiota induced by the different antibiotic treatments upon FMT in colitic animals, we performed a metataxonomic analysis of FMT recipients. The microbial community structures among groups were significantly different as measured by beta-diversity of Bray-Curtis dissimilarity, (PERMANOVA *p* < 0.05; Fig. 1b; Table S2) suggesting diverse microbial engraftments after FMT. The relative abundances of various taxa were different among groups (Fig. 1c). We observed higher levels of *Akkermansia*, *unclassified Bacteroidetes*, *Alistipes*, *Bifidobacterium*, and *Clostridia* (i.e., *Clostridium XI*, *Clostridiaceae1 uncl.*) in DSS^FMT^ mice. DSS^FMT + Streptomycin^ and DSS^FMT + Vancomycin^ animals showed the enrichment of the genera *Bacteroides*, *Parabacteroides*, *Streptococcus* as well as the reduction of other taxa among which *Bifidobacterium*, *Mucispirillum*, *unclassified Clostridiaceae1*, and *Clostridium XI*. Noteworthy, DSS^FMT + Metronidazole^ mice, that retained a full capability to control intestinal inflammation compared to DSS^FMT + Streptomycin^ and DSS^FMT + Vancomycin^ animals, showed a significant increase of the genus *Lactobacillus* (Fig. 1d). The gut microbiota was able to predict samples according to treatment among untreated colitic mice, normobiotic FMT-treated, and antibiotic pre-conditioned FMT-treated animals by using a Random Forest classifier. The most important features selected by the classifier were indeed among those differentially abundant among groups (Fig. 1d, e). With an out-of-bag (OOB) error rate of 29.5%, the classifier showed a moderate but significant prediction power (permutation tests *p* value = 0, accuracy = 69%, kappa = 43%). The classification error was 0.8% for DSS^FMT^ and 90% for DSS samples. These data collectively suggests that FMT is important to stabilize the gut microbiota during experimental colitis despite the inflammatory status of the host.

### Transfer of antibiotics preconditioned microbiota in DSS colitic recipients influences colonic iNKT and CD4^+^ T cell populations

The gut microbiota has a profound effect on the intestinal immune system functionality [1]. Therapeutic FMT administered during experimental colitis directly modulates mucosal immunity promoting IL10-dependent anti-inflammatory responses [12, 13].

**Fig. 2:**
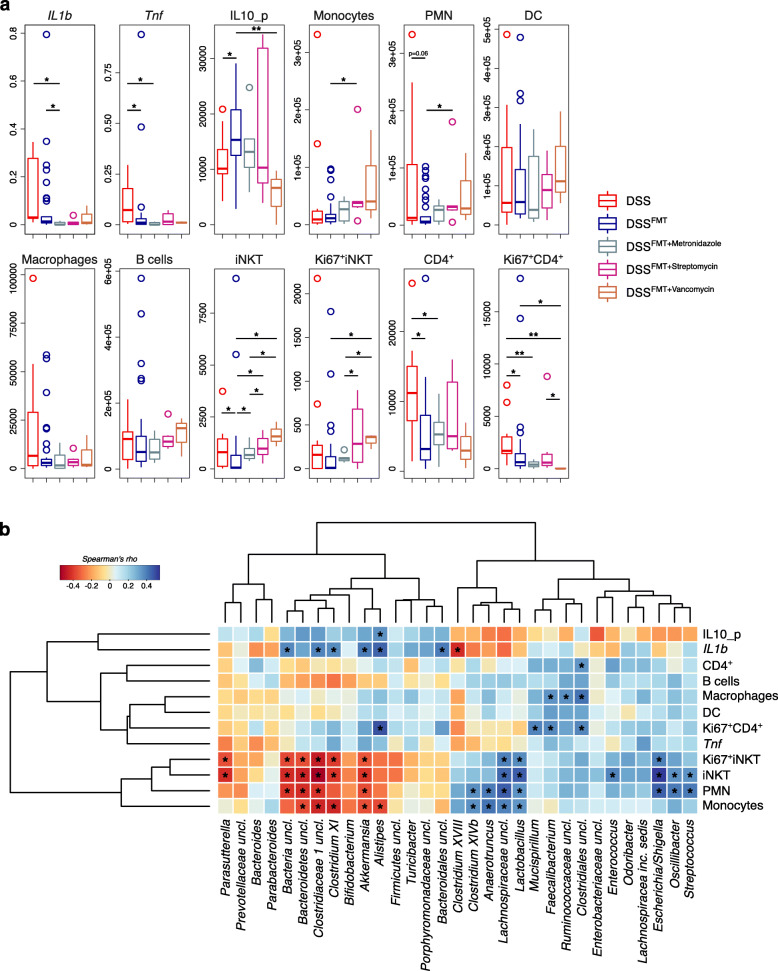
Immunophenotyping and co-occurrence of the gut microbiota with immune cell populations in DSS colitic recipients after FMT with antibiotics pre-conditioned microbiota. **a** Inflammation markers levels and absolute abundances of innate and adaptive immune cells isolated from the colonic lamina propria of DSS, DSS^FMT^, DSS^FMT + Metronidazole^, DSS^FMT + Streptomycin^, and DSS^FMT + Vancomycin^ animals. *p < 0.05, **p < 0.01, *** < 0.001; Wilcoxon rank-sum test. **b** Heatmap of Spearman’s *ρ* (ρs) correlations between the relative abundance of the most represented bacterial genera (with relative abundance > 0.1%) in the gut microbiota of colitic animals after treatments with the indicated immunological parameters. The significant correlations with an FDR-corrected *p* < 0.05 are indicated with an asterisk (*)

Thus, we analyzed the colonic immune cell landscape of FMT-treated animals in relation to the gut microbiota composition (Fig. 2). The presence/absence of specific bacterial taxa in the gut has been linked to the differentiation and expansion of conventional [34] and unconventional CD4^+^ T cells [35]. Accordingly, we observed an increase in the numbers of total and proliferating iNKT and CD4^+^ T cells in control colitic animals and FMT recipients treated with a gut microbiota altered by antibiotics preconditioning (Fig. 2a). In particular, DSS mice showed a significant enrichment of CD4^+^ and CD4^+^Ki67^+^ T cells compared to normobiotic FMT-treated animals. On the other hand, antibiotic preconditioning of the gut microbiota induced the significant increase of LP iNKT cells compared to normobiotic FMT recipients. Furthermore, DSS^FMT + Streptomycin^ and DSS^FMT + Vancomycin^ mice were characterized by the enrichment of proliferating Ki67^+^iNKT cells compared to DSS^FMT^ and DSS^FMT + Metronidazole^ animals (Fig. 2a). We then evaluated which bacterial taxa might be important to polarize mucosal immunity toward inflammation by correlating the inflammatory and immunophenotypic data with the gut microbiota (Fig. 2b). The microbial hallmarks of DSS^FMT^ mice, i.e., *Akkermansia*, together with *Clostridium X* and unclassified genera of *Bacteroidetes* and *Clostridiaceae1*, negatively correlated with monocytes, PMN, and iNKT cells. On the other hand, different pro-inflammatory taxa such as *Streptococcus* and *Escherichia/Shigella* positively correlated with PMN and both iNKT and Ki67^+^ iNKT cells. Also, the genus *Lactobacillus* correlated with the abundance of LP iNKT cells (Fig. 2b). These data suggest that different members of the gut microbiota modulate the abundance of pro- and anti-inflammatory immune cell populations dictating the success of FMT treatments to control inflammation.

### Antibiotics treatments shape the gut microenvironment and microbiota-derived metabolites of DSS colitic recipients

**Fig. 3:**
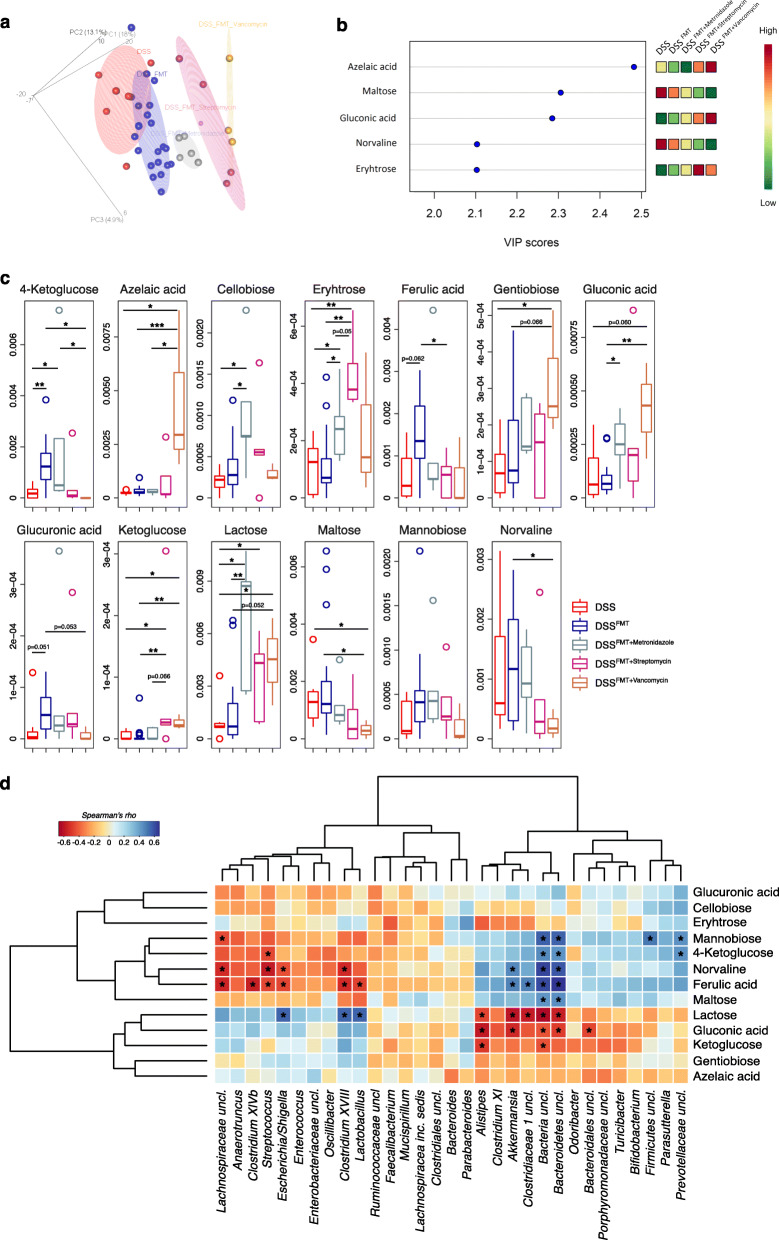
Metabolomics analysis and co-occurrence of the gut microbiota with microbiota-derived metabolites in DSS colitic recipients after FMT with antibiotics pre-conditioned microbiota. **a** Partial least square discriminant analysis showing clustered samples according to the type of antibiotic treatment. **b** Metabolites that differentiate samples according to treatment with a variable important in projection (VIP) score > 2. **c** Metabolites with the highest discriminative power for the classification of samples (VIP score > 2) according to treatment. *p < 0.05, **p < 0.01, *** < 0.001; Wilcoxon rank-sum test. **d** Heatmap of Spearman’s *ρ* (ρs) correlations between the relative abundance of the most represented bacterial genera (with relative abundance > 0.1%) in the gut microbiota of colitic animals after treatments with the metabolites with the highest discriminatory power. The significant correlations with an FDR-corrected *p* < 0.05 are indicated with an asterisk (*)

The phylogenetic composition of the gut microbiota is much more dynamic than community metabolism, as a consequence of microbial functional redundancy [36]. Given the relevant biological implications for host-microbiome interactions, in order to identify microbiota-derived metabolites important to modulate intestinal inflammation, we performed an untargeted metabolomics analysis of fecal samples in colitic animals upon FMT (Fig. 3). We observed significantly different metabolic profiles among groups (Fig. 3a) with azelaic acid, maltose, gluconic acid, norvaline, and erythrose being the metabolites with the highest discriminatory power (Fig. 3b). Higher levels of azelaic and gluconic acids were detected in DSS^FMT + Vancomycin^ mice as well as of erythrose in DSS^FMT + Streptomycin^ mice (Fig. 3c). The gut metabolome was also able to cluster samples according to their inflammation status (i.e., inflamed vs non-inflamed independently by the treatment) (Figure S1a and b) while the gut microbiota failed to do so (Figure S1c). Nevertheless, we have been able to identify bacterial taxa usually associated with inflammatory conditions and IBD, e.g., *Escherichia/Shigella* and *Fusobacterium* in inflamed vs non-inflamed samples (Figure S1d). We then correlated metabolomics and gut microbiota data observing the significant correlation of ferulic acid, gluconate, lactose, and norvaline with different bacterial taxa among which *Akkermansia*, *Bacteroidetes uncl.*, *Clostridium XVIII*, *Escherichia/Shigella*, and *Lactobacillus* (Fig. 3d). These data suggest that the transfer of different configurations of the gut microbiota affected by antibiotics enrich the gut microenvironment with metabolites that may dictate the status of intestinal inflammation.

### Exposure to a vancomycin-conditioned gut microenvironment skews human CD4^+^ T cells population toward a Th1/Th17 phenotype while metronidazole promotes IL10 secretion by iNKT cells

**Fig. 4:**
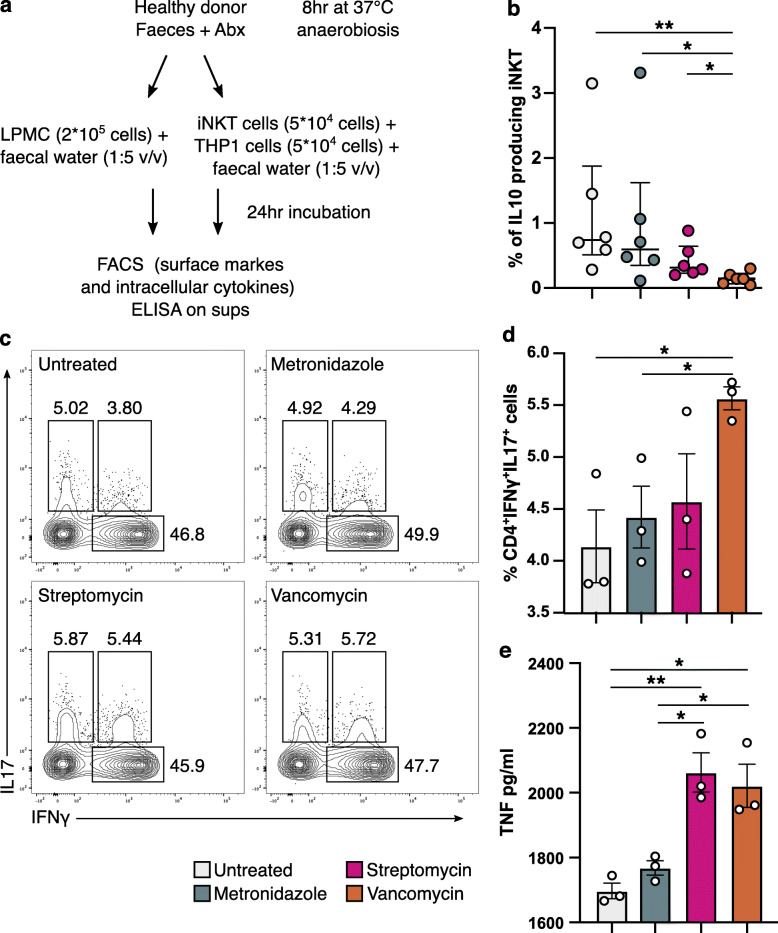
Ex vivo LPMC and iNKT cells stimulation assay with sterile filtered fecal water from antibiotic pre-conditioned human healthy donor’s microbiota. **a** Schematic representation of the experiment. **b** Frequencies of IL10 producing iNKT cells upon exposure to sterile fecal water from antibiotic pre-conditioned healthy donor’s microbiota. *p < 0.05, **p < 0.01, Mann-Whitney *U* test. **c** Representative contour plots. **d** Frequencies of IFNγ and IL17 secreting CD4^+^ T-cells from UC LPMC upon exposure to sterile fecal water from antibiotic preconditioned healthy donor’s microbiota. **e** TNF secretion of UC LPMC upon exposure to sterile fecal water from antibiotic preconditioned healthy donor’s microbiota. *p < 0.05, **p < 0.01, unpaired *t* test. One representative experiment out of at least two is shown

**Fig. 5:**
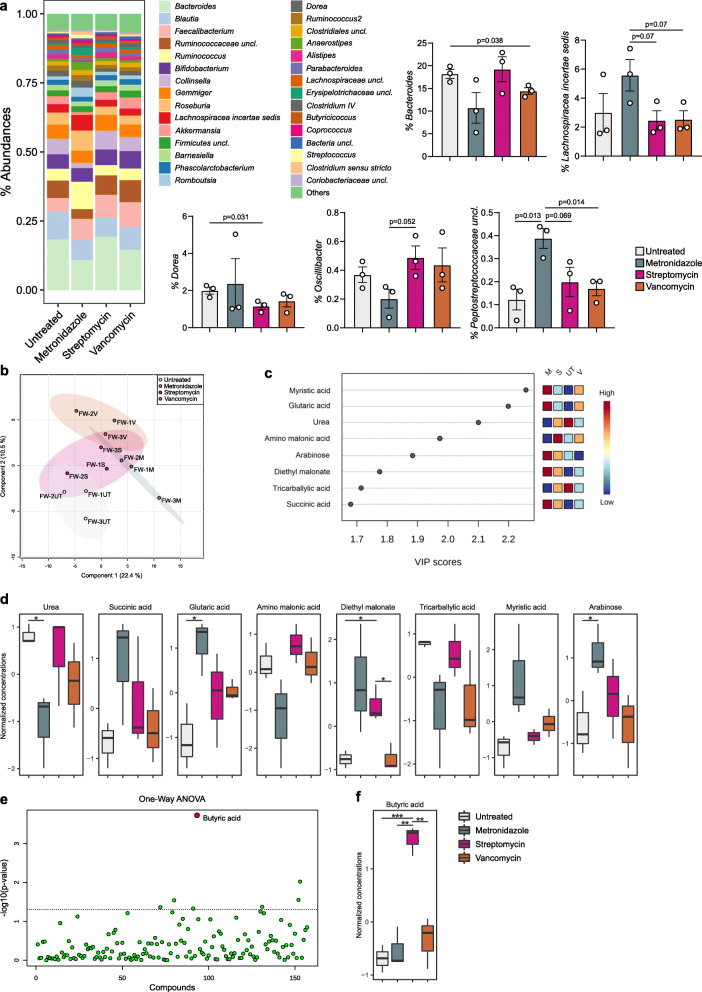
Gut microbiota composition and metabolomics analysis of the antibiotics-conditioned human fecal samples used for the ex vivo experiments. **a** Mean relative abundance at genus level of the antibiotics-conditioned human gut microbiota. All genera with relative abundance < 0.5% are reported together and labeled as “others.” **b** Significantly different taxa identified in the antibiotics-conditioned human gut microbiota. Exact *p* values are shown; unpaired *t* test. **c** Partial least square discriminant analysis showing clustered fecal water samples according to the type of antibiotic treatment. **d** Metabolites that differentiate samples according to treatment by variable important in projection (VIP) scores. **e** Normalized concentrations of metabolites with the highest discriminative power (VIP score > 1.5) for the classification of samples according to the type of treatment. **f** One-way analysis of variance; red dots indicate the significant metabolites (*p* < 0.05) after FDR-correction. **g** Normalized concentration of butyric acid as measured in FW samples. *p < 0.05, **p < 0.01, *** < 0.001; unpaired *t* test

Based on our previous data, we hypothesized that the metabolic products of gut microbiota may have an important role on the polarization of the host’s immune responses to modulate inflammation. Therefore, we performed an ex vivo experiment in which we exposed human colonic lamina propria mononuclear cells (LPMC) of ulcerative colitis patients to sterile fecal water (FW) from antibiotic pre-conditioned human healthy fecal microbiota to mimic the colonic microenvironment of our in vivo model (Fig. 4a). According to our hypothesis, we observed the skewing of CD4^+^ T cells toward a Th1/Th17 phenotype in the LPMC exposed to the FW from vancomycin-treated microbiota (Fig. 4c and d) as well as significantly higher levels of TNF production compared to metronidazole and untreated (antibiotic-free) FW. TNF levels were significantly increased also upon exposure to streptomycin FW (Fig. 4e). To note, monocytes and APC were not affected by FW treatments. Since iNKT cells were significantly affected by the antibiotics-conditioned microbiota in our in vivo colitis model, we performed an in vitro assay by exposing human intestinal iNKT cell clones isolated from IBD patients [28] to FW. We observed the polarization of iNKT cells toward the production of IL10 upon exposure to the metronidazole-conditioned FW as well as from the untreated FW (Fig. 4b). Interestingly, the microbial composition of the human fecal microbiota after antibiotic exposure (Figure 5a) showed few differences involving principally the genera *Bacteroides*, *Lachnospiracea incertae sedis*, *Dorea*, *Oscillibacter*, and *Peptostreptococcaceae* (Fig. 5b). The metabolomic analysis of the FW samples on the contrary showed distinct metabolic profiles according to the type of antibiotic treatment (Fig. 5c) with some highly discriminant metabolites deriving from amino acid catabolism, e.g., glutaric acid, succinate, amino malonate, and urea (Fig. 5d, e). Notably, we observed the significant enrichment of butyric acid in the streptomycin-conditioned FW (Fig. 5f, g), a possible indication for reactive oxygen species (ROS) production and accumulation of 8-oxoG lesions [37–39] despite the role of butyrate in regulating homeostatic immune responses [40, 41].

## Discussion

Breakdown of the complex interplay between the host immune system and the microbiota increases the risk of pathogen infection, the overgrowth of harmful pathobionts, and inflammatory disease. Although remarkable results have been obtained in understanding the role of the gut microbiota in different human pathologies and on the efficacy of therapies such as FMT and cancer immunotherapy [42, 43], there are paucity of data regarding the mechanism of action for the control of inflammation and the role that different commensals may have on intestinal immune responses. Here, we investigated how modifications of the gut microbiota by different antibiotic treatments affect the disease outcome in a model of acute experimental colitis upon FMT. We further expanded our results paralleling the findings observed in the animal model in a human setting by using colon biopsies from ulcerative colitis patients. We demonstrate that the transfer of an antibiotic-conditioned gut microbiota in colitic animals has an important role for the disease outcome and for the modulation of the inflammatory status, achieved by polarizing the immune response of the FMT recipient and modifying the gut microenvironment. In particular, the colitic recipients transferred with a streptomycin- or vancomycin-conditioned microbiota were characterized by blooming of the genera *Bacteroides*, *Parabacteroides*, *Streptococcus*, and unclassified *Enterobacteriaceae*, all associated with poor outcomes in human clinical studies evaluating FMT efficacy in IBD patients [44–46] as well as by the depletion of health-promoting bacteria, i.e., *Akkermansia* and *Bifidobacterium* [47, 48]. The gut microenvironment associated with these dysbiotic microbial communities showed the specific enrichment of metabolites that are abundant in the mouse gut after antibiotic treatments leading to susceptibility to *Clostridioides difficile* infections, i.e., gluconic acid [49], or during steatohepatitis, i.e., azelaic acid [50]. The immunological landscape of the recipients transferred with a vancomycin- or streptomycin-conditioned microbiota showed an increased infiltration of proinflammatory polymorphonuclear leukocytes and monocytes as well as proliferating iNKT cells in the intestinal LP. Since colonic APC levels and Ki67^+^iNKT cells have been shown to contribute to IBD pathogenesis [6, 13, 28, 51, 52], this could explain the inability of these microbiota configurations to control inflammation. Several reports described also the pivotal role of T cells as key players in the initiation and maintenance of intestinal inflammation through the recognition of bacterial antigens in IBDs [1]. Accordingly, exposure of LPMC from ulcerative colitis patients to vancomycin- and streptomycin-conditioned gut microenvironments (i.e., sterile fecal water) enriched with microbiota-derived metabolites polarized T cells toward a proinflammatory Th1/Th17 phenotype and the production of mediators of systemic inflammation, i.e., TNF. This effect could be mediated by the antibiotic-induced alteration of bacterial metabolism, since short-term exposure of healthy human microbiota to antibiotics caused alterations of amino acid catabolic products and enrichment of butyric acid. Although microbiota-derived butyrate is an important mediator of intestinal immune homeostasis [40, 41], other studies suggest that the gut microbiota, partially through an altered production of butyrate, induces ROS and the accumulation of 8-oxoG lesions, therefore contributing to inflammation and tumorigenesis [37–39]. On the other hand, the recipients that received FMT with a metronidazole-conditioned microbiota were able to control inflammation by reducing colitis severity. We hypothesize that this effect may be in part mediated by the genus *Lactobacillus*. Indeed, lactobacilli have a long history of use as health-promoting bacteria and several studies supported their role in the maintenance of intestinal homeostasis [53, 54]. The recipients receiving the metronidazole-conditioned microbiota showed the enrichment of cellobiose, a metabolite boosting the intestinal levels of *Lactobacillus* and *Bifidobacterium* [55]. Nevertheless, cellobiose is also used as a marker of intestinal permeability in Crohn’s disease patients [56]. *Lactobacillus* and *Bifidobacterium* promotes the release of anti-inflammatory IL10 by APC and T cells, which is pivotal for the resolution of intestinal inflammation [57] by reducing the proliferative capacity of proinflammatory T cells [13]. Also, iNKT cells produce IL10 in murine models of inflammatory disease upon therapeutic FMT [13]. Accordingly, the exposure of human-isolated iNKT cell clones to metronidazole-conditioned fecal water promoted the secretion of IL10, supporting the hypothesis that metronidazole shapes the microbiota and the gut microenvironment favoring a better control of colitis severity and inflammation compared to streptomycin and vancomycin.

## Conclusions

Taken together, our findings confirm that the ability of the gut microbiota to control inflammation is in part mediated by the production of microbiota-derived metabolites. Alterations of the gut microbiota induced by different antibiotic treatments, such as with vancomycin and streptomycin, polarize the immune system toward a pro-inflammatory configuration ablating any benefits of FMT. Metronidazole treatment, on the contrary, allowed the retention of a beneficial microbiota that reduced the severity of colitis suggesting that not all antibiotic treatments are associated with generalized detrimental effects on host-microbes interactions.

## Supplementary Information

**Additional file 1: Figure S1.** Gut Microbiota composition and metabolomics analysis of DSS colitic animals after FMT with antibiotics pre-conditioned microbiota analysed according to their inflammation status. a) Partial Least Square discriminant analysis showing clustered samples according to the inflammation status. b) Metabolites that differentiate samples according to the inflammation status with a Variable Important in Projection (VIP) score > 2. c) Beta-diversity analysis on Bray-Curtis dissimilarity (p=0.459, PERMANOVA) d) Most discriminant bacterial taxa identified by LEfSe analysis. Positive and negative LDA scores indicate taxa enriched or depleted in the gut microbiota of samples grouped based on their inflammation status. Only taxa having a *p*<0.05 (Wilcoxon rank-sum test) and LDA>|2.0| are shown.

**Additional file 2: Table S1.** FACS antibodies and dyes.

**Additional file 3: Table S2.** PERMANOVA of beta-diversity analysis as measured by Bray-Curtis dissimilarity.

**Additional file 4: Table S3.** Sample metadata, unrarefied OTU table and taxonomic classifications.

## Data Availability

16S rRNA gene sequencing raw data are available in SRA Repository with accession number PRJNA494680 and in the European Nucleotide Archive with accession number PRJEB41434. Sample metadata, unrarefied OTU table, and taxonomic classifications are available in the Supplementary Table S3.

## Abbreviations

ABX: Antibiotics
APC: Antigen-presenting cells
DSS: Dextran sodium sulfate
FMT: Fecal microbiota transplantation
IBD: Inflammatory bowel diseases
LP: Lamina propria
LPMC: Lamina propria mononuclear cells
Th: T-helper
