# Supplementary figures and images for: Antibiotic-associated dysbiosis affects the ability of the gut microbiota to control intestinal inflammation upon fecal microbiota transplantation in experimental colitis models
Source: Microbiome. 2021 Feb 6;9:39. doi: 10.1186/s40168-020-00991-x (PMC7868014; doi:10.1186/s40168-020-00991-x)

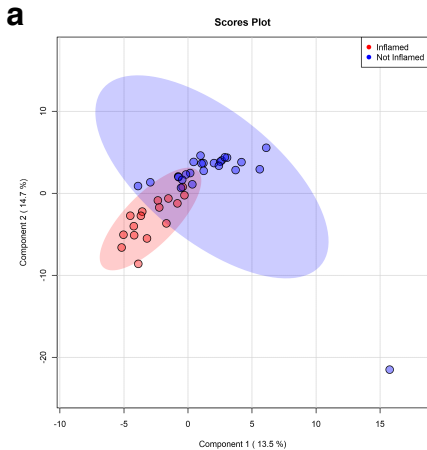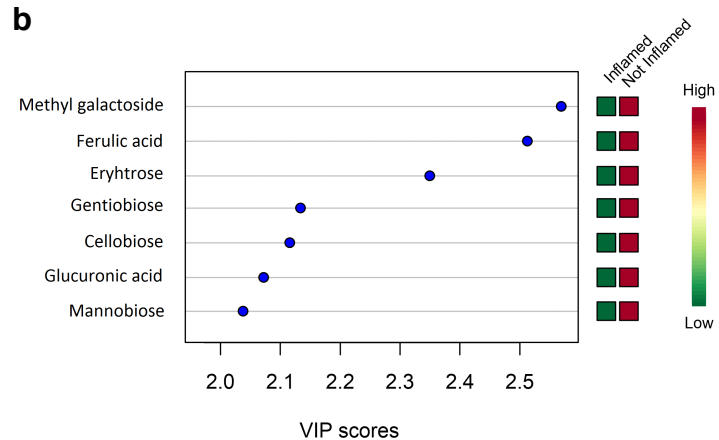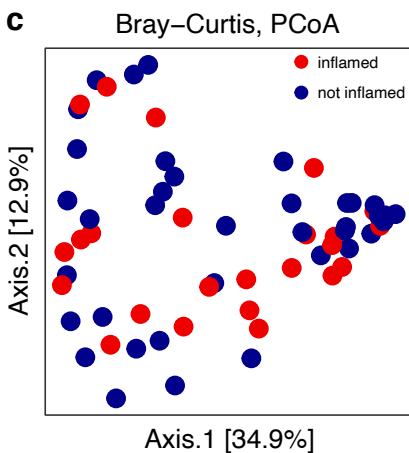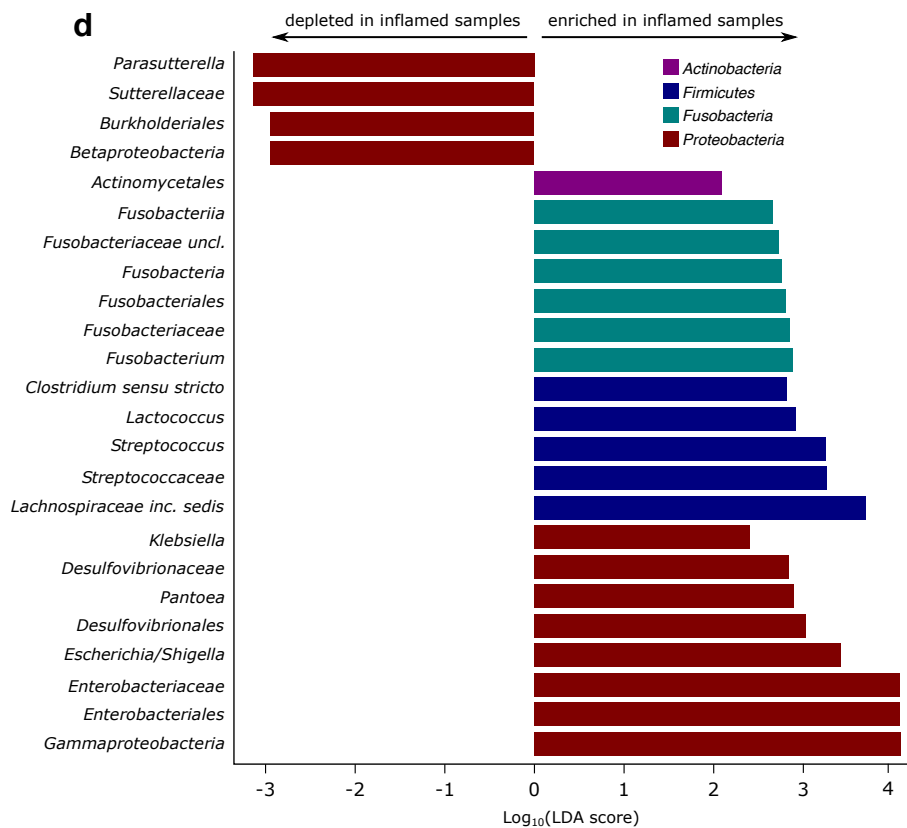

Supplement: Supplementary file 2 — Additional file 1: Figure S1. Gut Microbiota composition and metabolomics analysis of DSS colitic animals after FMT with antibiotics pre-conditioned microbiota analysed according to their inflammation status. a) Partial Least Square discriminant analysis showing clustered samples according to the inflammation status. b) Metabolites that differentiate samples according to the inflammation status with a Variable Important in Projection (VIP) score > 2. c) Beta-diversity analysis on Bray-Curtis dissimilarity (p=0.459, PERMANOVA) d) Most discriminant bacterial taxa identified by LEfSe analysis. Positive and negative LDA scores indicate taxa enriched or depleted in the gut microbiota of samples grouped based on their inflammation status. Only taxa having a p<0.05 (Wilcoxon rank-sum test) and LDA>|2.0| are shown. [file 40168_2020_991_MOESM2_ESM.pdf]
