# Supplementary material for: Antibiotic-associated dysbiosis affects the ability of the gut microbiota to control intestinal inflammation upon fecal microbiota transplantation in experimental colitis models
Source: Microbiome. 2021 Feb 6;9:39. doi: 10.1186/s40168-020-00991-x (PMC7868014; doi:10.1186/s40168-020-00991-x)
Supplement: Supplementary file 3 — Additional file 2: Table S1. FACS antibodies and dyes. [file 40168_2020_991_MOESM3_ESM.pdf]

Table S1: FACS antibodies and dyes

**mAbs**	**clone**	**Supplier**
CD45.2	104	Biolegend
CD3	17A2	BD
CD4	GK1.5	BD
CD11c	HL3	BD
CD19	1D3	BD
CD11b	M1/70	BD
F4/80	BM8	Biolegend
Ly6g	1A8	Biolegend
Ly6c	AL-21	eBioscience
MHC-II	M5/114.15.2	eBioscience
Ki67	16A8	Biolegend
**hAbs**	**clone**	**Supplier**
CD3	UCHT1	TONBO
CD4	SK3	eBioscience
CD11c	3.9	Biolegend
HLA-DR	LN3	eBioscience
CD14	M5E2	Biolegend
IL10	JES3-9D7	eBioscience
IL17A	eBio64DEC17	eBioscience
INFg	4S.B3	BD
Tetramer		NIH
Zombie Dye		Biolegend
