# Supplementary material for: Antibiotic-associated dysbiosis affects the ability of the gut microbiota to control intestinal inflammation upon fecal microbiota transplantation in experimental colitis models
Source: Microbiome. 2021 Feb 6;9:39. doi: 10.1186/s40168-020-00991-x (PMC7868014; doi:10.1186/s40168-020-00991-x)
Supplement: Supplementary file 4 — Additional file 3: Table S2. PERMANOVA of beta-diversity analysis as measured by Bray-Curtis dissimilarity. [file 40168_2020_991_MOESM4_ESM.pdf]

Table S2: PERMANOVA of beta-diversity analysis as measured by Bray-Curtis dissimilarity

	**F**	**R^2**	***p*-value**
DSS *vs* DSS^{FMT}	2.404786	0.051822	0.028
DSS *vs* $DSS^{FMT+Metronidazole}$	3.575931	0.192503	0.003
DSS *vs* $DSS^{FMT+Streptomycin}$	2.890872	0.161584	0.004
DSS *vs* $DSS^{FMT+Vancomycin}$	2.147415	0.151788	0.038
DSS^{FMT} *vs* $DSS^{FMT+Metronidazole}$	7.579476	0.162721	0.001
DSS^{FMT} *vs* $DSS^{FMT+Streptomycin}$	6.226671	0.137677	0.001
DSS^{FMT} *vs* $DSS^{FMT+Vancomycin}$	4.798523	0.117615	0.003
$DSS^{FMT+Metronidazole}$ *vs* $DSS^{FMT+Streptomycin}$	1.8961	0.15939	0.052
$DSS^{FMT+Metronidazole}$ *vs* $DSS^{FMT+Vancomycin}$	2.766451	0.283261	0.014
$DSS^{FMT+Streptomycin}$ *vs* $DSS^{FMT+Vancomycin}$	2.268719	0.244772	0.013
